# Protein structure protection commits gene expression patterns

**DOI:** 10.1186/gb-2008-9-7-r107

**Published:** 2008-07-07

**Authors:** Jianping Chen, Han Liang, Ariel Fernández

**Affiliations:** 1 Program in Applied Physics, Rice Quantum Institute, Rice University, Houston, TX 77005, USA; 2 Department of Ecology and Evolution, University of Chicago, Chicago, IL 60637, USA; 3 Department of Bioengineering, Rice University, Houston, TX 77005, USA; 4 Department of Computer Science, University of Chicago, Chicago, IL 60637, USA

## Abstract

**Background:**

Gene co-expressions often determine module-defining spatial and temporal concurrences of proteins. Yet, little effort has been devoted to tracing coordinating signals for expression correlations to the three-dimensional structures of gene products.

**Results:**

We performed a global structure-based analysis of the yeast and human proteomes and contrasted this information against their respective transcriptome organizations obtained from comprehensive microarray data. We show that protein vulnerability quantifies dosage sensitivity for metabolic adaptation phases and tissue-specific patterns of mRNA expression, determining the extent of co-expression similarity of binding partners. The role of protein intrinsic disorder in transcriptome organization is also delineated by interrelating vulnerability, disorder propensity and co-expression patterns. Extremely vulnerable human proteins are shown to be subject to severe post-transcriptional regulation of their expression through significant micro-RNA targeting, making mRNA levels poor surrogates for protein-expression levels. By contrast, in yeast the expression of extremely under-wrapped proteins is likely regulated through protein aggregation. Thus, the 85 most vulnerable proteins in yeast include the five confirmed prions, while in human, the genes encoding extremely vulnerable proteins are predicted to be targeted by microRNAs. Hence, in both vastly different organisms protein vulnerability emerges as a structure-encoded signal for post-transcriptional regulation.

**Conclusion:**

Vulnerability of protein structure and the concurrent need to maintain structural integrity are shown to quantify dosage sensitivity, compelling gene expression patterns across tissue types and temporal adaptation phases in a quantifiable manner. Extremely vulnerable proteins impose additional constraints on gene expression: They are subject to high levels of regulation at the post-transcriptional level.

## Abstract 2

A proteomic association study between protein three-dimensional structure and transcriptional and post-transcriptional regulation in yeast and human.

## Background

The coordination of protein roles to achieve specific biological functions requires the spatial/temporal concurrence of proteins so that they can form complexes [1,2] or, in general, operate within a module [2-4]. In turn, this concurrence is tightly coordinated through the regulation of gene expression, as suggested by established correlations between the transcriptome and the interactome [5,6]. However, structure-encoded factors that may quantitatively control such correlations have not been identified. So far, protein structure has not provided organizing clues for the integration of large-scale descriptions of the molecular phenotype.

As reported in this work, by exploiting a structure-based analysis of protein associations [7,8] and their correlated expression patterns, we identify a structural attribute, protein vulnerability, and show that it commits gene expression patterns in a quantifiable manner. More specifically, protein vulnerability is shown to determine the extent of co-expression of genes containing protein-encoding interactive domains in metabolic adaptation phases [9,10] or tissue types [11,12], while extreme vulnerability promotes significant post-transcriptional regulatory control.

Soluble proteins maintain the integrity of their functional structures provided the amide and carbonyl groups paired through hydrogen bonds are adequately shielded from water attack, preventing backbone hydration and, generally, the concurrent total or partial denaturation of the soluble structure [13,14]. As shown in this work, this integrity is often ensured through the formation of protein complexes, which become more or less obligatory depending on the extent of structure vulnerability and the level of backbone protection provided by the association [13]. By adopting vulnerability as a structural indicator of dosage imbalance effects, the extent of reliance on binding partnerships is precisely quantified and shown to be an organizing factor for the yeast and human transcriptome.

## Results

### Protection of a vulnerable protein and co-expression demands

**Figure 1:**
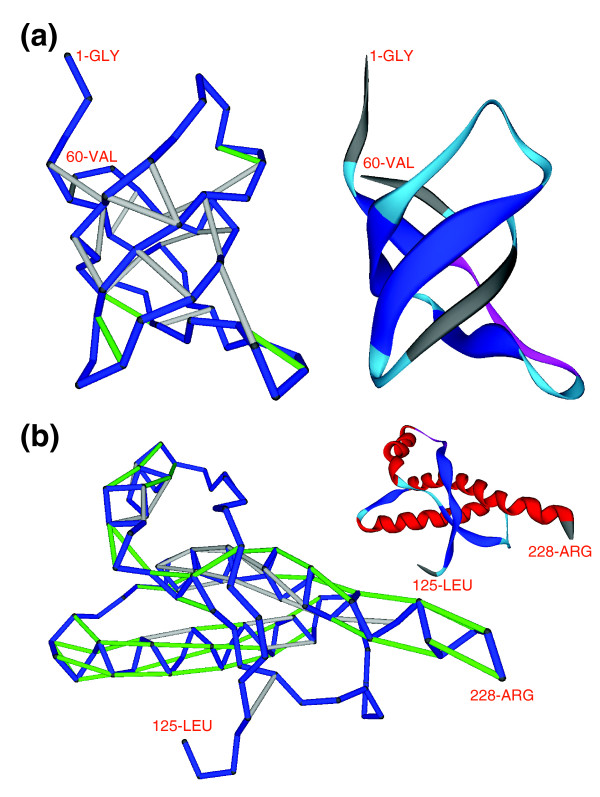
Hydrogen-bond pattern and structural vulnerabilities (SEBHs) of the yeast SH3 domain and the human prion protein PrP^C^. **(a) **Hydrogen-bond pattern and structural vulnerabilities (SEBHs) of the yeast SH3 domain from a *S. cerevisiae *40.4 kDa protein (PDB.1SSH) [17]. The ribbon display is included as a visual aid. The protein backbone is shown as virtual bonds (blue) joining consecutive α-carbons in the peptide chain. Light-grey segments represent well protected backbone hydrogen bonds, and green segments represent SEBHs. The extent of solvent-exposure extent of a hydrogen bond was determined from atomic coordinates by calculating the number of nonpolar groups within its microenvironment (Materials and methods). SEBHs are those backbone hydrogen bonds protected by an insufficient number of nonpolar groups as statistically defined in Materials and methods. The level of structure vulnerability *ν*, defined as the ratio of SEBHs to the overall number of backbone hydrogen bonds, is 19.0% (*ν *= 4/21). **(b) **SEBH-pattern for the cellular structure of the human prion protein PrP^C ^(PDB.1QM0) [18]. Its vulnerability parameter is *ν *= 63.0%, making it the most vulnerable soluble folder of all structures of unbound proteins reported in the PDB.

We start by defining vulnerability *ν *of a soluble protein structure as the ratio of solvent-exposed backbone hydrogen bonds (SEBHs) to the overall number of such bonds (Figure 1). The SEHBs may be computationally identified from atomic coordinates (Materials and methods). Thus, while backbone hydrogen bonds are determinants of the basic structural motifs [15,16], the SEHBs represent local weaknesses of the structure.

**Figure 2:**
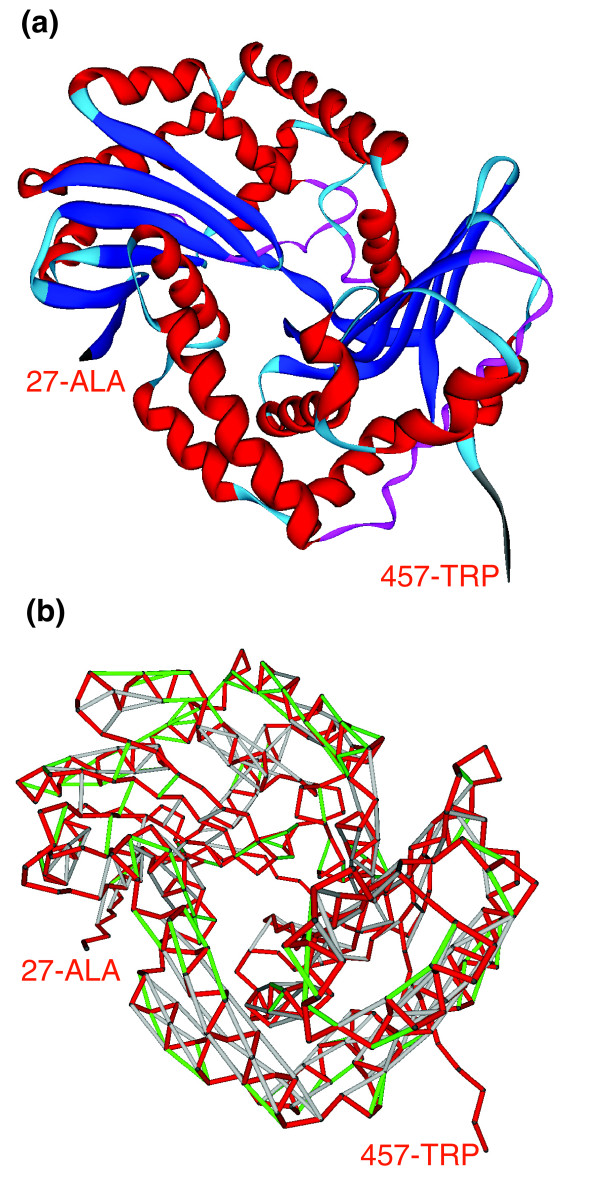
Ribbon representation and vulnerability (SEBH) pattern of subunit 1 from the cytochrome b-c1 complex. **(a) **Ribbon representation and **(b) **vulnerability (SEBH) pattern of subunit 1 from the cytochrome b-c1 complex (PDB.1KB9) [19]. In b, red segments represent virtual protein backbone bonds, light-grey segments represent well protected backbone hydrogen bonds, and those green segments represent SEBHs. In the cytochrome complex, this protein adopts a highly vulnerable (*ν *= 57.3%) conformation.

Figure 1a shows the vulnerability pattern of a well protected soluble protein, the yeast SH3 signaling domain [17], with *ν *= 19.0%. Figure 1b shows the most vulnerable protein structure for an autonomous folder in the Protein Data Bank (PDB) (*ν *= 63.0%), the cellular form of the 90-230 fragment of the human prion protein PrP^C ^(PDB.1QM0) [18]. This extreme case was detected after exhaustive computation of the *ν *parameter for all conformations of isolated (those not in a complex) polypeptide chains reported in the PDB (Materials and methods). Figure 2 shows the most vulnerable structure adopted by a protein chain within a yeast complex: subunit 1 from the cytochrome b-c1 complex (*COR1/YBL045C*). Unlikely to be found in isolation, this structure is found within the mitochondrial respiratory chain complex III [19].

A vulnerable soluble structure gains extra protection of its backbone hydrogen bonds through forming complexes, as nonpolar groups of a binding partner contribute to expel water molecules from the microenvironment of the preformed bonds [13]. On the other hand, the SEBHs promote their own dehydration as a means to stabilize and strengthen the hydrogen bond [14].

To delineate the role of structure vulnerability as an organizing integrative factor in large-scale descriptions of the molecular phenotype, we first examined the Pfam-filtered [7] protein complexes for yeast [8] and human [20]. These associations involve domains whose PDB-reported homologs are involved in complexes.

This work quantitatively examines the relationship between the structural vulnerability of a protein and the extent of co-expression of genes encoding its binding partners. Thus, the extent of co-expression, *η *(*i*, *j*), for two genes *i*, *j *encoding interacting proteins is measured by the expression correlation of the two genes normalized to the average correlation over the interactome (Materials and methods). In consonance, the expression correlation of a complex, *η (complex)*, may be defined by the maximum expression correlation over its constitutive underlying pairwise interactions (see Additional data files 7-9 for alternative definitions).

**Figure 3:**
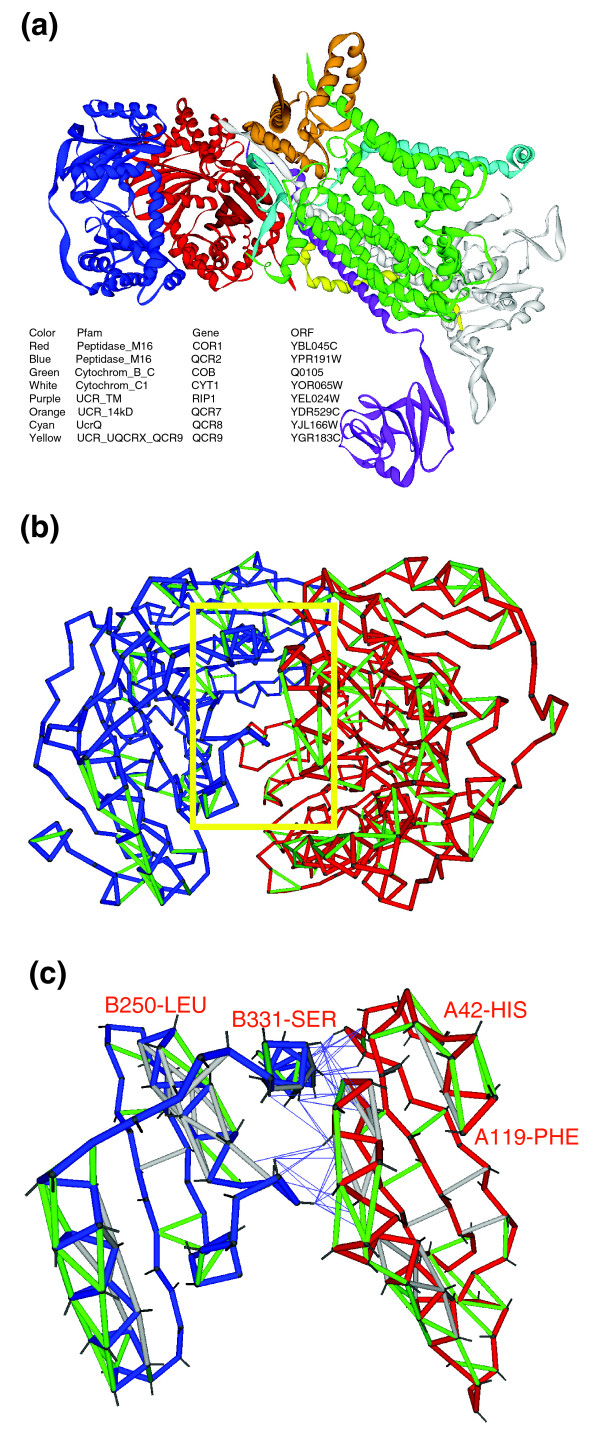
Mutual protection of SEBHs in the two subunits of mitochondrial respiratory chain complex III. **(a) **Ribbon representation of mitochondrial respiratory chain complex III (PDB.1KB9). The high structure vulnerability of subunit 1 (red; compare Figure 2) renders it highly needy for interaction with other subunits of the complex to maintain its structural integrity. **(b) **SEBH pattern for subunit 1 (red) and subunit 2 (blue). The interacting pair is characterized by a very high expression correlation *η *= 3.61. The yellow square highlights the part of the interface shown in detail in (c). **(c) **Illustration of mutual protections of SEBHs in the two subunits along part of their interface. One side-chain bond (between α and β carbons) is displayed. The thin blue lines, which connect β-carbons in one protein with centers of hydrogen bonds in the other protein, represent mutual protections of hydrogen bonds across the protein-association interface. Thus, a thin line is shown whenever the side chain of one protein is contributing with nonpolar groups to the microenvironment of a preformed hydrogen bond in its binding partner.

Thus, the most highly correlated yeast complex (overall *η (complex) *= 3.61) with full PDB-reported representation is the mitochondrial respiratory chain complex III shown in Figure 3a (PDB.1KB9[19]). The most vulnerable protein within the complex (*ν *= 57%) is subunit 1 from the cytochrome b-c1 complex (Gene/ORF = *COR1/YBL045C*, shown in red). Its peptide chain conformation, with the SEBH pattern described in Figure 2, is involved in the most highly correlated interaction (*η *= 3.61) within the complex (Figure 3b,c). The binding partner in this interaction is subunit 2 of cytochrome b-c1 (Gene/ORF = *QCR2/YPR191W*, blue chain in Figure 3a). Figure 3c shows the mutual protection of preformed SEBHs in the two subunits along part of their association interface (red, *COR1 *residues 42-119; blue, *QCR2 *residues 250-331). This intermolecular mutual 'wrapping' of local weaknesses illustrates the fact that the association contributes to maintain structural integrity (Figure 3c).

**Figure 4:**
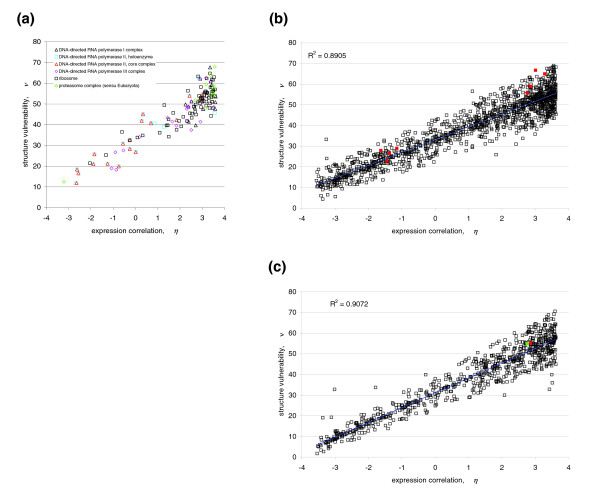
Correlation between maximum structure vulnerability *ν *and co-expression similarity *η *for yeast protein interactions. **(a) **Correlation between maximum structure vulnerability *ν *and co-expression similarity *η *for interactions within specific yeast complexes. The *ν*-parameter of an interaction is defined as the maximum vulnerability between the two interacting partners, and the *η*-parameter is the ratio of their expression correlation to the (non-zero) expected correlation over all interacting pairs in the proteome. **(b) **(*η*-*ν*) correlation for all Pfam-filtered yeast protein interactions. Red points represent interactions involving extremely vulnerable proteins, including confirmed yeast prions (Additional data file 5). **(c) **(*η*-*ν*) correlation of Pfam-filtered yeast protein interactions involving only PDB-reported proteins. The red data point represents an interaction involving an extremely vulnerable protein, and the green point represents an interaction involving an extremely vulnerable protein reported to be a prion protein (ERF2) [24-26].

We examined the role of structure vulnerability as a factor governing the extent of co-expression of binding partners in illustrative yeast complexes (Figure 4a; Additional data file 1). Structure-based protein-protein interactions were curated through the Pfam database, so that two proteins were considered to interact with each other if their respective domains (or homolog domains) were reported in a PDB complex [8,21]. The expression correlation, *η*, for each interaction pair within a complex was determined at the mRNA level of the genes whose open reading frames (ORFs) contained the interacting domains (Materials and methods). Vulnerabilities were computed either directly from PDB files, when available, as described in Figure 1, or from atomic coordinates generated by homology threading using the Pfam-homolog domain as template (Materials and methods). In the latter case, side-chain equilibration, constrained by a fixed homology-threaded backbone, was obtained from constrained molecular dynamics simulations (Materials and methods). We then determined the maximum *ν*-value for each interactive pair and, using the comprehensive microarray database for *Saccharomyces cerevisiae *glucose→ glycerol metabolic adaptation [22], we computed the expression correlation *η *for each Pfam interaction. A tight *(η-ν) *correlation (R^2 ^= 0.891) is obtained and shown to hold across the illustrative yeast complexes (Figure 4a) and, furthermore, to hold across all 1,354 pairs of interacting proteins in the yeast interactome with Pfam representation (Figure 4b,c; Additional data file 2). The *(η-ν) *correlation implies that the protection of a functionally competent protein structure in yeast drives co-expression of its binding partners to an extent that is determined by the structure vulnerability.

In selecting the yeast transcriptome [22], particular attention was focused on the 'perturbative' nature of the change triggering the structural remodeling of the proteomic network across different phases. A more extensive remodeling on a vastly larger scale, as in the complete yeast developmental cycle [23], cannot be treated as a perturbation since it clearly alters the modular structure of the proteome network [4] and, consequently, yields a weaker *(η-ν) *correlation (Additional data file 10).

**Figure 5:**
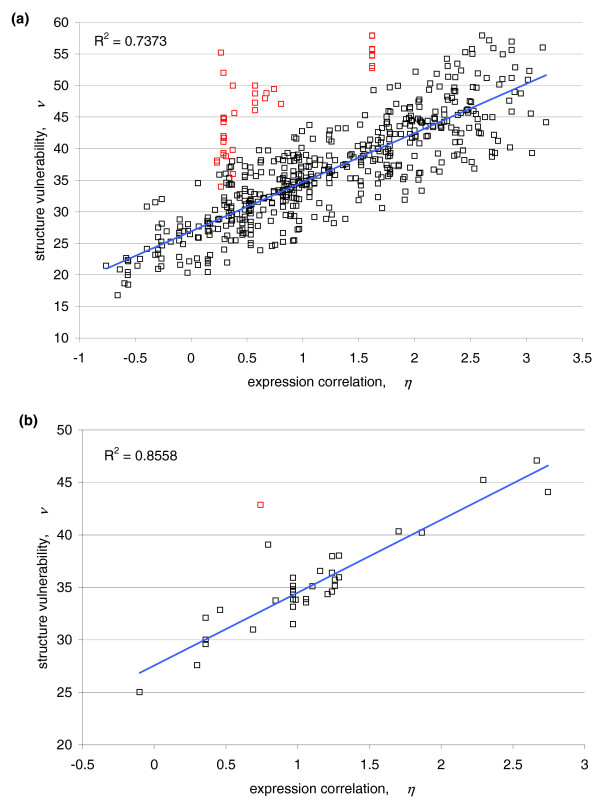
(*η *- *ν*) correlation for human protein interactions. **(a) **The (*η*-*ν*) correlation for all Pfam-filtered human protein interactions. Red points represent interactions involving extremely vulnerable proteins (Additional data file 4). **(b) **The correlation over Pfam-filtered human protein interactions that involve only PDB-reported proteins. The red point represents an interaction containing an extremely vulnerable protein.

Structure vulnerability is not only an organizing factor for the metabolic-adaptation transcriptome but also steers the organization of tissue-based transcriptomes. This is revealed by a similar comparative analysis of the most comprehensive protein-encoding gene-expression data for human [11] and the structure-represented interactome [20]. Thus, a clear *(η-ν) *correlation is apparent between the co-expression of 607 gene pairs and the maximum structure vulnerability for each pair of interacting domains encoded in the ORFs of the respective genes (Figure 5; Additional data file 3).

Other human transcriptomes based on normal tissue expression were examined (see, for example, [24]), but none provided statistically significant (>>10 genes pairs) representation for the gene pairs for which interactome data also exist [20], as needed for the present study.

### Post-transcriptional regulation of the expression of highly vulnerable proteins

In contrast with the tighter yeast correlation, a few but significant outlier pairs (Figure 5, red data points) are found beyond the confidence band defined by a width of two Gaussian dispersions from the linear *(η-ν) *fit. To rationalize this fact, we identified 115 human genes with ORFs encoding extremely vulnerable proteins (Additional data file 4). Consistent with the definition of structure vulnerability (Figure 1), the latter proteins are identified by large sequences (≥ 30 residues) of amino acids that are poor protectors of backbone hydrogen bonds. In principle, a sizable window of residues unable to protect backbone hydrogen bonds produces a poor folder, yielding a highly vulnerable structure [14,25]. Thus, these sequences are either probably unable to sustain a stable soluble structure, or prone to relinquish the folding information encoded in the amino acid sequence in favor of self-aggregation [25]. The poor protectors (G, A, S, Y, N, Q, P) are amino acids possessing side chains with insufficient nonpolar groups, with polar groups too close to the backbone (thus precluding hydrogen-bond protection through clustering of nonpolar groups) [14] or with amphiphilic aggregation-nucleating character (Y) [26-28]. Charged backbone de-protecting side chains (D, E) are excluded since they would entail negative design relative to protein self-aggregation. All outlier interactions in the human *(η-ν) *correlation involve genes with extreme vulnerability (Figure 5; Additional data file 4). Significantly, when the same criterion for extreme vulnerability is used to scan the yeast genome (Additional data file 5), 85 genes are identified whose ORFs encode the five confirmed prion proteins for this organism [26-29]: PSI+ (*SUP35*), NU+ (*NEW1*), PIN+ (*RNQ1*), URE3 (*URE2*) and SWI+ (*SWI1*). This fact is statistically significant (*P *< 10^-10^, hypergeometric test) and supports the presumed relationship between structural vulnerability of the soluble fold and aggregation propensity [25].

The *(η-ν) *correlation reported in Figure 5 for human is weaker than the yeast counterpart likely because, in contrast with yeast, mRNA levels are not a reliable surrogate for protein expression levels in human [30,31]. This observation led us to examine post-transcriptional regulation in human genes, to analyze the microRNA (miRNA) targeting of the predicted 115 extremely vulnerable human genes (Additional data files 4 and 6), and to contrast the miRNA-targeting statistics with the generic values across the human genome [31]. To obtain statistics on miRNA targeting, we identified putative target sites in the 3' UTR (untranslated region) of each gene for 162 conserved miRNA families (Materials and methods) [31]. Thus, 7,927 out of 17,444 genes (45.4%) are predicted to contain at least one miRNA target site (Additional data file 6), while 87 out of 105 (82.9%) extremely vulnerable genes are predicted to be targeted genes. Thus, human genes containing extremely vulnerable regions are more frequently targeted by miRNA (*P *<< 1.31 × 10^-5^, binomial test). In regards to miRNA regulation complexity, the mean number of miRNA target sites for human genes is 2.66 and the median is 0, while the mean number for extremely vulnerable genes is 6.01 and the median is 5. This significant difference (*P *< 10^-16^, Wilcox rank test) strongly suggests that the deviation of extremely vulnerable genes from the *(η-ν) *correlation (Figure 5), with expression correlation evaluated at the level of mRNA expression, can be explained by post-transcriptional miRNA regulation. This type of regulation influences the final protein expression level. In a broad sense, this analysis highlights the connection between protein structure and gene regulation: extremely vulnerable genes require tight control at the post-transcriptional level.

### Protein intrinsic disorder and transcriptome organization

The inability of an isolated protein fold to protect specific intramolecular hydrogen bonds from water attack may lead to structure-competing backbone hydration with concurrent local or global dismantling of the structure [14,25,32]. This view of structural vulnerability suggests a strong correlation between the degree of solvent exposure of intramolecular hydrogen bonds and the local propensity for structural disorder [33-35]: in the absence of binding partners, the inability of a protein domain to exclude water intramolecularly from pre-formed hydrogen bonds may be causative of a loss of structural integrity, and this tendency is marked by the disorder propensity of the domain [32]. These findings led us to regard the predicted extent of disorder in a protein domain as a likely surrogate for its vulnerability and to contrast it with the extent of expression correlation with its interactive partners. The disorder propensity may be determined by a sequence-based score, *f*_*d*_(*f*_*d *_= 1, certainty of disorder; *f*_*d *_= 0, certainty of order), assigned to each residue. In this work, this parameter is generated by the highly accurate predictor of native disorder PONDR-VSL2 [34,35]. The extent of intrinsic disorder of a domain may be defined as the percentage of residues predicted to be disordered relative to a predetermined *f*_*d *_threshold (*f*_*d *_= 0.5).

**Figure 6:**
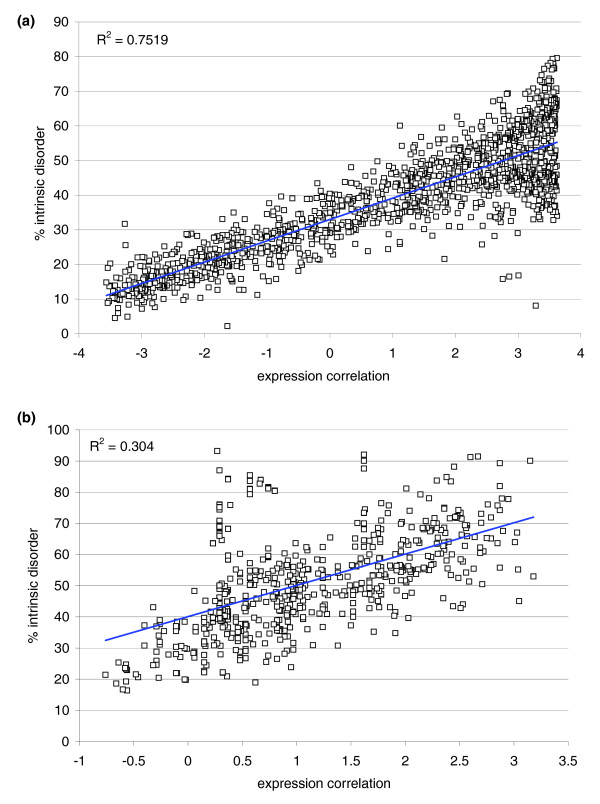
*(η*-disorder) correlation for yeast and human protein interactions. Correlation between *η*-parameter and percent predicted disorder (disorder content) of the most disordered domain for each of **(a) **the 1,354 Pfam-filtered protein-interaction pairs in yeast and **(b) **the 607 pairs in human.

Reexamination of the expression correlations in the yeast and human transcriptomes was carried out, taking into account a proteome-wide sequence-based attribution of the extent of disorder (percentage of residues predicted to be disordered, or 'disorder content') in interacting protein domains. The disorder predictions did not include any structural information on induced fits arising upon forming a complex, and hence, unlike structure vulnerability, the percent predicted disorder is independent of the complex under consideration. This fact introduces deviations in the estimation of vulnerability through disorder content for proteins with extensive disorder content since their conformational plasticity may enable diverse induced-fit conformations with different vulnerabilities (Figure 6a). In yeast, the extent of disorder of the most disordered domain for each pair of interacting domains captures the degree of correlation in the expression patterns required for structure protection (Figure 6a). This is revealed by the correlation between the extent of disorder of the most disordered domain in an interacting pair and the expression correlation *η *of the two genes encoding the respective interacting domains. While weaker than the *η*-*ν *correlation (Figure 4), the *η*-disorder correlation is still relatively strong for yeast proteins (R^2 ^= 0.752; Figure 6a), implying that disorder content determines the degree of coexpression of binding partners to a significant extent. The large dispersion in disorder extent at high levels of coexpression (approximately 45% dispersion versus approximately 15% for proteins with low disorder/low expression correlation) is indicative that highly disordered chains may adopt structures with very different levels of vulnerability depending on the complex in which they are involved (the *η*-*ν *correlation does not widen so significantly for smaller *η*-values). Thus, the more disordered the chain, the more multi-valued the correspondence between disorder extent and vulnerability, conferring higher dispersion to the *η*-disorder correlation.

The *η*-disorder correlation in human is considerably weaker (R^2 ^= 0.304; Figure 6b) than in yeast. This is partly due to the fact that human proteins have a higher degree of disorder propensity than their yeast orthologs [36] and, hence, they are capable of significantly diversifying their structural adaptation (induced folding) in different complexes. In this context, the extent of disorder becomes a poor surrogate of structural vulnerability, as different ν-values may correspond to a single percent predicted disorder. In addition, post-transcriptional regulation in humans implies that expression correlations at the mRNA level are not reflective of the protein concurrencies modulated by tissue type, as indicated above.

To conclude, Figure 6 reveals the role of intrinsic protein disorder in transcriptome organization suggested by exploring the interrelationship between protein vulnerability and disorder propensity.

## Discussion

Soluble protein structures may be more or less vulnerable to water attack depending on their packing quality. As shown in this work, one way of quantifying the structure vulnerability is by determining the extent of solvent exposure of backbone hydrogen bonds. Within this scheme, local weaknesses in the protein structure may become protected upon forming a complex, as exposed backbone hydrogen bonds become exogenously dehydrated. Vulnerable structures are thus quantitatively reliant on binding partnerships to maintain their integrity, suggesting that vulnerability may be regarded as a structure-based indicator of gene dosage sensitivity [37,38]. This observation is validated by establishing the significance of protein vulnerability or structure protection as an organizing factor in temporal phases (yeast) and tissue-based (human) transcriptomes. Specifically, this role was established by examining the degree of co-expressions of a protein with its binding partners in structure-represented interactions. Thus, for each Pfam-filtered binding partnership, the extent of co-expression across metabolic adaptation phases (yeast) or tissue types (human) was found to depend quantitatively on the structure vulnerability of the proteins involved. Hence, vulnerability may be regarded as an organizing factor encoded in the structure of gene products.

Furthermore, as shown in this work, the tight coordination between translation regulation and gene function dictates that extremely vulnerable, and hence 'highly needy', proteins are subject to significant levels of post-transcriptional regulation. In human, this extra regulation is achieved through extensive miRNA targeting of genes coding for extremely vulnerable proteins. In yeast, on the other hand, our results imply that such a regulation is likely achieved through sequestration of the extremely vulnerable proteins into aggregated states. Intriguingly, the 85 yeast genes encoding extremely vulnerable proteins included those for the five confirmed yeast prions [26-29]. This statistically significant result implies that if the extremely vulnerable proteins are themselves translational regulators, this sequestration may directly lead to epigenetic consequences and phenotypic polymorphism [26-28].

## Conclusion

In this work we adopted a structural biology perspective to reassess the fundamental notion of 'dosage imbalance effect' and examine the implications for gene expression, specifically for transcriptomal organization and post-transcriptional regulation. Thus, vulnerability of protein structures and the concurrent need to maintain structural integrity for functional reasons prove to be quantifiers of dosage imbalance: proteins with a high degree of reliance on binding partnerships to maintain their structural integrity are naturally expected to yield high dosage sensitivity in their respective gene expressions. Hence, structural vulnerability is shown to be a determinant of transcriptome organization across tissues and temporal phases: the need for protein structure protection compels gene co-expression in a quantifiable manner. Extreme vulnerability is shown to require significant additional regulation at the post-transcriptional level, manifested by epigenetic aggregation in yeast and miRNA targeting in human. These latter observations will likely inspire further study of structure-encoded signals that govern post-transcriptional regulation.

## Materials and methods

### Expression data sources

Yeast expression data were obtained from the comprehensive *Saccharomyces *Genome Database [22]. This complete dataset contains mRNA expression levels during a transition from glucose-fermentative to glycerol-based respiratory growth. Human expression data were taken from the comprehensive Novartis Gene Expression Atlas [11]. This dataset includes 158 array images composed of 79 samples, each of which has two replicates hybridized on the human genome HG-U133A array. We discarded six samples of cancer tissues: ColorectalAdenocarcinoma, leukemialymphoblastic(molt4), lymphomaburkittsRaji, leukemiapromyelocytic, lymphomaburkittsDaudi, and leukemiachronicmyelogenous (k562).

### Interaction data sources

Protein interaction curation based on structure provides direct physical interactions [8]. Two proteins were considered to interact with each other when their respective domains or homologs of their respective domains were found in a complex with PDB-reported structure. We obtained curated yeast protein domain interactions from the Structural Interaction Network [8], and filtered them using recently published yeast interaction data [21]. For human, we focused on interactions within complexes. The complex data were obtained from the MIPS/Mammalian Protein Complex Database [20]. We used the protein domain descriptions in the Pfam database [7], and searched for domain-domain interactions using iPfam [39].

### Expression correlation η

The expression correlation for a protein-protein interaction is a normalized quantity defined as the Pearson correlation of the expression vectors of the genes encoding for the interacting domains divided by the mean correlation over all gene pairs encoding for interacting domains. The normalization is necessary for comparative analysis across species because different species have different mean expression correlations and, hence, the significance of a correlation is necessarily a relative attribute. Given its statistical nature, the denominator is non-zero for any species since, in a statistical sense, protein pairs that interact are expected to be positively correlated in their expression. We use the Pearson correlation coefficients of expression vectors to determine similarity between expression profiles. For two expression vectors **X **and **Y**, the Pearson correlation coefficient Corr(**X**, **Y**) is given by:

$$\text{Corr}(\text{X},\text{Y})=\frac{<(X-<X>)(Y-<Y>)>}{\sqrt{<X^{2}>-<X{>}^{2}}\sqrt{<Y^{2}>-<Y{>}^{2}}}$$

where *X*, *Y *are generic coordinates in the vectors ***X ***and ***Y***, respectively, and < > indicates mean over the 73 normal tissues (human) [11] or over the 5 metabolic adaptation phases (yeast) [22].

### Calculation of vulnerability ν and identification of SEBHs for soluble proteins

To determine the extent of solvent exposure of a backbone hydrogen bond in a soluble protein structure, we determine the extent of bond protection from atomic coordinates. This parameter, denoted ρ, is given by the number of side-chain nonpolar groups contained within a desolvation domain (hydrogen-bond microenvironment) defined as two intersecting balls of fixed radius (the approximate thickness of three water layers) centered at the α-carbons of the residues paired by the hydrogen bond. In structures of PDB-reported soluble proteins, at least two-thirds of the backbone hydrogen bonds are protected on average by ρ = 26.6 ± 7.5 side-chain nonpolar groups for a desolvation ball radius of 6 Å. Thus, SEBHs lie in the tails of the distribution, that is, their microenvironment contains 19 or fewer nonpolar groups, so their ρ-value is below the mean (ρ = 26.6) minus one standard deviation (= 7.5).

In cases where the protein structures were unavailable from the PDB, we generated atomic coordinates through homology threading adopting the Pfam homolog as template and using the program Modeller [40-42]. Modeller is a computer program that models three-dimensional structures of proteins subject to spatial constraints [40], and was adopted for homology and comparative protein structure modeling. We thus generate the alignment of the target sequence to be modeled with the Pfam-homolog structure reported in the PDB and the program computes a model with all non-hydrogen atoms. The input for the computation consists of the set of constraints applied to the spatial structure of the amino acid sequence to be modeled and the output is the three-dimensional structure that best satisfies these constraints. The three-dimensional model is obtained by optimization of a molecular probability density function with a variable target function procedure in Cartesian space that employs methods of conjugate gradients and molecular dynamics with simulated annealing.

### Homolog PDB sources

Yeast PDB homologs were obtained from the *Saccharomyces *Genome Database [43], and human PDB homologs were from Pfam [44].

### Micro-RNA targeting analysis

For 17,444 human genes, we identified putative target sites for 162 conserved miRNA families using TargetScanS (version 4.0), a leading target-prediction program [45]. Thus, we obtained the number of target-site types in the 3' UTR of each gene [31]. Among the genes in our analysis: 105 genes were identified as encoding extremely vulnerable proteins; 7,927 out of 17,444 genes (45.4%) are predicted to be miRNA targets (containing at least one type of miRNA target site); and 87 out of 105 genes encoding extremely vulnerable proteins (82.9%) are predicted to be target genes. Thus, genes encoding extremely vulnerable proteins tend to be miRNA target genes (*P *<< 1.31 × 10^-5^, binomial test).

In terms of miRNA regulation complexity, the average number of miRNA target-site types for a human gene is 2.66 and the median number is 0; while the average number for a prion gene is 6.01 and the median is 5. Again, this is highly significant (*P *< 10^-16^, Wilcox rank test).

### Prediction of native disorder of protein domains

The highly accurate predictor of native disorder PONDR VSL2 [34,35] exploits the length-dependent (heterogenous) amino acid compositions and sequence properties of intrinsically disordered regions to improve prediction performance. Unlike previous PONDR predictors for long disordered regions (>30 residues), it is applicable to disordered regions of any length. The disorder score (*0 *≤ *f*_*d *_≤ *1*) is assigned to each residue within a sliding window, representing the predicted propensity of the residue to be in a disordered region (*f*_*d *_= *1*, certainty of disorder; *f*_*d *_= *0*, certainty of order). The disorder propensity is quantified by a sequence-based score that takes into account residue attributes such as hydrophilicity, aromaticity, and their distribution within the window interrogated.

## Abbreviations

miRNA, micro RNA; ORF, open reading frame; PDB, Protein Data Bank; SEBH, solvent-exposed backbone hydrogen bonds; UTR, untranslated region.

## Authors' contributions

JC provided theoretical insight, designed methodology, generated and collected data, and co-wrote the paper. HL provided theoretical insight, and generated and collected data. AF provided the fundamental concepts and insights, designed methodology and wrote the paper.

## Additional data files

The following additional data are available with the online version of this paper. Additional data file 1 provides raw data for Figure 4a. Additional data file 2 provides Raw data for Figure 4b,c. Additional data file 3 provides raw data for Figure 5. Additional data file 4 lists extremely vulnerable proteins in human. Additional data file 5 lists extremely vulnerable yeast proteins. Additional data file 6 lists the predicted number of miRNA targets for human genes. Additional data file 7 outlines the robustness of results with respect to alternative graph-theoretic definitions of co-expression similarity. Additional data file 8 outlines how vulnerability correlates with co-expression similarity in protein complexes. Additional data file 9 provides Raw data: yeast (a) and human (b) complexes examined in Additional data file 8. Additional data file 10 shows the (η-ν) plot obtained for the yeast developmental-phase transcriptome obtained from a comprehensive identification of cell cycle-regulated genes by microarray hybridization [23].

## Supplementary Material

Additional data file 1 Data in column A indicate the expression correlation *η *associated with protein interactions, and data in column B indicate the structure vulnerability *ν *for interactions within specific complexes. The rest of the columns contain the ORF, domain and structure information (PDB accession code of interacting domain or its Pfam-homologs), respectively, for every pair of interacting proteins. Click here for file

Additional data file 2 Sheet 1 contains all Pfam-filtered yeast protein interactions, while sheet 2 contains only those interactions with both partners having PDB structures. In each sheet, column A lists the expression correlation h of interactions, and columns B and C list the structure vulnerability n of interactions not involving or involving, respectively, extremely vulnerable proteins. The remaining columns contain ORF, domain and structure information (PDB accession code of interacting domain or of its Pfam-homologs) for every pair of interacting proteins. Click here for file

Additional data file 3 Sheet 1 contains all Pfam-filtered human protein interactions, while sheet 2 contains only those interactions with both partners having PDB structures. In each sheet, column A contains the expression correlation h for each interaction, and columns B and C list the structure vulnerability n of interactions not involving or involving, respectively, extremely vulnerable proteins, and the rest of the columns list gene name, protein ID, domain and structure information (PDB accession code of interacting domain or of its Pfam-homologs) of every pair of interacting proteins. Click here for file

Additional data file 4 The extremely vulnerable proteins in human are identified from genome-wide scanning of protein-encoding regions with sequence windows (length ≥ 30) containing mainly amino acids (G, A, S, Y, N, Q, P) that are poor protectors of the protein backbone. An extremely vulnerable protein contains at least one such window with a threshold of three amino acids allowed to be outside the group of poor protectors. Click here for file

Additional data file 5 Extremely vulnerable yeast proteins are determined in the same way as for human (Additional data file 4). The rows marked in green correspond to the five confirmed yeast prions [26-29]: SUP35 (ERF2), URE2, NEW1, RNQ1 and SWI1. Click here for file

Additional data file 6 The number of putative target-site types corresponding to 162 conserved miRNA families determined for 17,444 human genes by interrogation of the 3' UTR using TargetScanS (version 4.0) [45]. Click here for file

Additional data file 7 The co-expression similarity for genes *i*, *j*, encoding a pair of interacting proteins is alternatively measured as the adjacency *a*_*ij*_(β) = (0.5 + 0.5 *η *(*i*, *j*))^β^, where *η *(*i*, *j*) is the expression correlation for the gene pair *i*, *j *and β is a soft threshold [46]. Similarly, the structure vulnerability is alternatively defined as ν_*i*, *j*_*(β) *= ν (*i*, *j*)^β^, where ν (*i*, *j*) is the maximum ν-value for the interacting pair. **(a, b) **(ν *(β)*-*a(β)) *correlations for yeast for exponents β = 0.5 (a) and 10 (b). The adjacencies for β = 1 correspond simply to a linear rescaling of η already correlated with ν in Figure 4. **(c, d) **The same as (a, b) but for human. Notice that high exponents (β > 1) tend to amplify differences in co-expression, yielding lower correlation coefficients (R^2 ^in (ν *(β)*-*a(β)) *plots). Click here for file

Additional data file 8 A normalized co-expression similarity *γ (β, complex) *for all genes encoding proteins that form a complex is obtained from the adjacencies of the pairwise interactions within the complex as: *γ (β, complex) *= [median_i, j ∈ complex_*a*_*ij*_(β)]/median_i, j _*a*_*ij*_(β)], where the median in the denominator extends over all interactive pairs in the interactome. Similarly, the normalized structure vulnerability Λ *(β, complex) *for complexes is defined as Λ *(β, complex) *= [median_i, j ∈ complex_*ν*_*ij*_(β)]/median_i, j _*ν*_*ij*_(β)]. **(a-c) **(Λ *(β, complex)-γ (β, complex)) *correlation over all 98 yeast complexes with transcriptome representation for exponents β = 0.5 (a), 1 (b) and 10 (c). **(d-f) **The same as (a-c) but for 53 human complexes. Click here for file

Additional data file 9 **(a) **Yeast complexes. **(b) **Human complexes. Click here for file

Additional data file 10 (*η*-*ν*) plot obtained for the yeast developmental-phase transcriptome obtained from a comprehensive identification of cell cycle-regulated genes by microarray hybridization [23] Click here for file
